# Dicarbonyl stress and mitochondrial dysfunction in the aged heart

**DOI:** 10.18632/aging.204704

**Published:** 2023-05-01

**Authors:** Diana Bou-Teen, Elisabet Miro-Casas, Marisol Ruiz-Meana

**Affiliations:** 1 Cardiovascular Diseases Research Group, Vall d’Hebron Institut de Recerca (VHIR), Vall d’Hebron Hospital Universitari, Vall d’Hebron Barcelona Hospital Campus, Barcelona,Spain; 2 Centro de Investigación Biomédica en Red de Enfermedades Cardiovasculares (CIBER-CV), Barcelona, Spain

**Keywords:** aging, cardiomyocytes, advanced glycation-end products, mitochondria, FoF1-ATP synthase

In the heart, more than 90% of the daily consumed ATP is produced by oxidative phosphorylation at the inner membrane of mitochondria. About 70–80% of this ATP is used to fuel muscle contraction and to regulate the calcium necessary for such cyclic mechanical activity. Therefore, mitochondrial ATP generation must be finely tuned to calcium-dependent contraction in cardiomyocytes, which is sustained by a tightly regulated communication between mitochondria and sarcoplasmic reticulum (SR), in which the ATP generated by mitochondria allows cell relaxation in diastole (i.e., SR-mediated calcium uptake), whereas calcium released through the ryanodine receptor (RyR) in the SR during systole regulates the rate of mitochondrial ATP generation. One of the earliest phenotypic traits of the aged heart is the energy demand/supply mismatch that underlies its reduced tolerance to exercise and lower adaptability to stress. This energy insufficiency manifests at the level of its most important functional unit, the cardiomyocytes. Although the causes of such energy inefficiency may be several, some studies have documented a defective RyR and VDAC bridging that results in impaired SR-mitochondrial anatomo-functional coupling [1]. This defect in the interorganelle communication results in altered calcium transfer from SR to mitochondria, which in turn contributes to reduced antioxidant regeneration and increased pro-oxidative status at the SR-mitochondria microenvironment [2].

Aging is characterized by a progressive loss of molecular fidelity due to a time-dependent reduction in the efficacy of genetically-encoded cell repairing mechanisms. The paradigm of non-regulated molecular damage is the stochastic chemical attack of dicarbonyl compounds to proteins and other macromolecules. Dicarbonyl compounds are extremely reactive and ubiquitous alpha-oxaldehydes (methylglyoxal, glyoxal and 3-deoxyglucosone) that are generated as metabolic intermediates of glycolysis, gluconeogenesis and lipid metabolism. They spontaneously and preferentially react with positively charged amino acids within proteins forming heterogeneous and irreversible chemical adducts, collectively named as advanced glycation end-products, that can provoke modifications in the proteins’ tertiary and quaternary structure rendering them dysfunctional [3]. Dicarbonyl stress refers to a pathological state in which there is an abnormal endogenous accumulation of these compounds, either as a consequence of an increased generation or reduced detoxification [4]. The contribution of dicarbonyl stress to cell dysfunction during aging is raising a growing scientific interest, as it has been described in some of the most prevalent age-associated diseases, including neurodegenerative diseases, chronic renal failure, atherosclerosis and hypertension [4]. In addition, the increase in oxidative stress typical of aging interferes with the detoxification of dicarbonyl compounds, while the accumulation of dicarbonyls favors ROS production. Our group was a pioneer in documenting an abnormal accumulation of dicarbonyl-induced chemical damage in the myocardium of elderly humans (>75 years) and old mice (>20 months) driven by a deficient activity of the glyoxalase-mediated detoxification system [5]. Importantly, a significant fraction of the proteins targeted by such dicarbonyl species was found to be located at the intracellular compartment of cardiomyocytes [5].

The dicarbonyl stress present in the aged heart has been shown to have deleterious consequences on cardiac mitochondria through different mechanisms. On the one hand, the molecular damage of RyR induced by dicarbonyl species increases spontaneous calcium leak from SR, so that chronic exposure of surrounding mitochondria (mainly interfibrillar mitochondria) to a dysregulated calcium environment favors an excessive mitochondrial calcium uptake and accumulation, a compensatory response that leads to matrix calcium precipitation and damage. This mechanism has been described in human and murine cardiac mitochondria, and contributes to the reduced aerobic capacity of the heart during aging [1, 5]. On the other hand, dicarbonyl species can react with several amino acids within different subunits of mitochondrial ATP synthase, the molecular rotary machine that culminates oxidative phosphorylation [6]. The mitochondrial ATP synthase (or FoF1-ATP synthase) is a highly conserved and abundant protein present in all eukaryotic cells, whose role in cell death and survival goes beyond ATP generation. Hence, spontaneous dimerization/oligomerization of FoF1-ATP synthase monomers folds de mitochondrial inner membrane into cristae, amplifying the respiratory surface of the cells [7]. Nevertheless, under certain pathological conditions, such as ischemia, in which electron transport stops and the intermembrane H+ gradient dissipates, the FoF1-ATP synthase switches its mode of operation and converts into an energy consuming device (ATP hydrolase), precipitating ATP exhaustion. In addition, evidences from independent studies strongly support the concept that FoF1-ATP synthase could be the molecular entity of the mitochondrial permeability transition pore (mPTP), a stress-induced unspecific channel whose persistent opening causes an extreme form of energy dissipation [8].

By using high throughput mass spectrometry, our group identified five subunits within FoF1-ATP synthase that are targeted by dicarbonyl species in cardiomyocytes from aging mice, including subunits e and g, which are essential for dimerization and cristae folding [6]. Blue-native coupled to *in situ* enzyme assay confirmed that dicarbonyl modifications of FoF1-ATP synthase altered the ability of monomers to self-assemble into dimers/oligomers. Ultrastructural images of murine myocardium indicated that defective cardiac FoF1-ATP synthase dimerization/oligomerization is associated with the presence of aberrant cristae in some mitochondria (reduced tip curvature, onion-like mitochondria) that are absent in the myocardium of young mice [6]. As expected, suboptimal cristae morphogenesis was paralleled by less efficient oxidative phosphorylation in intact mitochondria despite no age-related changes in the protein expression of the different FoF1-ATP synthase subunits. By contrast, *in vitro* evaluation of ATPase activity using denaturalized mitochondria, in which cristae architecture does not have any contribution to energy efficiency, demonstrated that the enzyme activity was preserved in aging. Importantly, both mitochondria from aged mice and from H9c2 cells chronically exposed to dicarbonyl stress were found to have an increased susceptibility to undergo mPTP [6], an important cause of cell death during myocardial ischemia-reperfusion injury.

**Figure 1:**
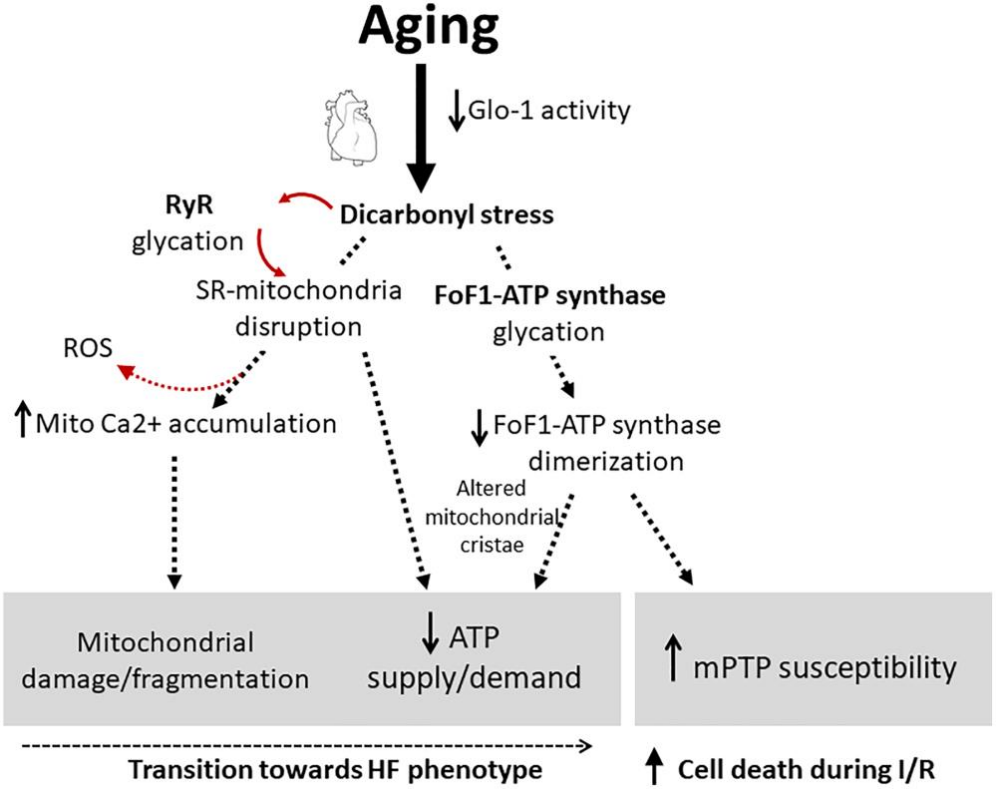
**Pathophysiological contribution of dicarbonyl stress to mitochondrial dysfunction in the heart during aging through the glycation of RyR and FoF1-ATP synthase.** Accumulation of dicarbonyl compounds is favored by the reduction in the efficiency of glyoxalase-dependent detoxification system. This mechanism underlies an energy supply/demand mismatch in the aging cardiomyocytes, favoring the transition towards a failing phenotype, and increases cells’ susceptibility to undergo mPTP and death during I/R injury. Abbreviations: Glo-1: glyoxalase 1; RyR: ryanodine receptor; SR: sarcoplasmic reticulum; mPTP: mitochondrial permeability transition pore; HF: heart failure; I/R: ischemia/reperfusion.

In summary, dicarbonyl stress favored by a deficient glyoxalase-dependent detoxification may represent a fundamental previously underestimated mechanism of mitochondrial damage and energy deficiency in the aged heart (Figure 1). Dicarbonyl-induced mitochondrial alteration secondary to molecular damage of some key proteins involved in calcium homeostasis (RyR) and ATP generation (FoF1-ATP synthase) can have important pathophysiological consequences during aging, facilitating the transition of a metabolically competent cardiomyocyte into a failing cell and aggravating cardiomyocyte necrosis during ischemia-reperfusion injury. Pharmacological tools addressed to scavenge dicarbonyl compounds or alleviate their functional consequences deserve further investigation.
